# Use of resources and costs associated with the treatment of Dupuytren’s contracture at an orthopedics and traumatology surgery department in Denia (Spain): collagenase clostridium hystolyticum versus subtotal fasciectomy

**DOI:** 10.1186/1471-2474-14-293

**Published:** 2013-10-14

**Authors:** Rafael Sanjuan Cerveró, Nuria Franco Ferrando, Jaime Poquet Jornet

**Affiliations:** 1 Orthopedics and Traumatology Surgery (OTS) Department of the Hospital de Denia, Denia, Alicante, Spain; 2 Chief of the Clinical Department of Pharmacy, Health Department in Denia, Denia, Alicante, Spain; 3 Hospital De Denia Marina Salud, Partida Beniadlà, S/N, Dénia, Alicante 03700, Spain

**Keywords:** Dupuytren, Collagenase Clostrodium histolyticum, Costs

## Abstract

**Background:**

Our purpose was to analyze and compare the use of direct health resources and costs generated in the treatment of Dupuytren's contracture using two different techniques: subtotal fasciectomy and infiltration with Collagenase Clostridium Histolyticum (CCH) in regular clinical practice at the Orthopedic and Traumatology Surgery (OTS) Department at the Hospital de Denia (Spain).

**Methods:**

Observational, retrospective study based on data from the computerized clinical histories of two groups of patients- those treated surgically using a one or two digit subtotal fasciectomy technique (FSC) and those treated with CCH infiltration, monitored in regular clinical practice from February, 2009 to May, 2012. Demographic (age, sex), clinical (number of digits affected and which ones) and use of resources (hospitalizations, medical visits, tests and drugs) data were collected. Resource use and associated costs, according to the hospital’s accounting department, were compared based on the type of treatment from Spain’s National Health Service.

**Results:**

91 patients (48 (52.8%) in the FSC group) were identified. The average age and number of digits affected was 65.9 (9.2) years and 1.33 (0.48) digits affected in the FSC group, and 65.1 (9.7) years and 1.16 (0.4) digits in the CCH group.

Overall, the costs of treating Dupuytren's disease with subtotal FSC amount to €1,814 for major ambulatory surgery and €1,961 with hospital stay including admission, surgical intervention (€904), examinations, dressings and physiotherapy. As to collagenase infiltration, costs amount to €952 (including minor surgery admission, vial with product, office examination and dressings). Finally, comparing total costs for treatments, a savings of €388 is estimated in favor of CCH treatment in the best-case scenario (patient under MAS system with no need for physiotherapy) and €1,008 in the worst-case scenario (patient admitted to hospital needing subsequent physiotherapy), implying a savings of 29% and 51%, respectively.

**Conclusions:**

This study demonstrates that treating patients with DC by injection with CCH at the OTS department of the Hospital de Denia generates a total savings of 29% and 51% (€388 and €1008) compared with fasciectomy at the time of treatment. Long term evolution of CCH treatment is uncertain and the recurrence rate unknown.

## Background

Fibrosis in the superficial palmar aponeurosis, causing the contracture of fingers in flexion, is known as Dupuytren’s disease. Although its description is attributed to Guillaume Dupuytren (1777–1835) after he published the first edition of Leçons Orales in 1832 [1], it had already been described by Astley Cooper [2]. Several factors are involved in its pathogenesis, among which are the differentiation of fibroblasts into myofibroblasts, local hyperemia, an increase of matrix protein synthesis (type III collagen and fibronectin), and the final contraction of these proteins, both at an intracellular and an extracellular level, which is responsible for this disease [3].

With variable prevalence and incidence (3-6%), it is associated with Caucasian population groups from Northern Europe [4,5]. There is no clear etiology of the pathology, although some predisposing factors are known: genetic factors, trauma, smoking, diabetes mellitus, aging, alcohol consumption, several drugs (phenobarbital, protease inhibitors, isocyanide) and the presence of free radicals. Once the disease is established, it demonstrates constant progression, although this is variable over time. Factors for early onset are family forms, young age at onset, concomitant presence of Ledderhose disease or Dupuytren’s diathesis, and Knuckle pads [6].

Currently, treatment establishes surgical and non-surgical approaches. Among the former, the treatment of choice is fasciectomy, used in 80-90% of primary surgery cases [4,5]. This is the most commonly used technique in Spain [7], although there are other approaches in use, including percutaneous cordectomy, Jacobsen-type flap [8], dermofasciectomy or even amputation, as a last option [6]. Non-surgical options [9] include needle aponeurotomy or intralesional injection of various substances, such as corticosteroids, fibrinolysin, pepsin, trypsin, hyaluronidase or thiomucase, all of which are aimed at degrading collagen after infiltration of the cord and show unsatisfactory clinical results.

Collagenase Clostridium Histolyticum (CCH) is an exception to this last group, since it acts on the level of degradation of types I and III collagen through two different subtypes of enzymes belonging to the metalloprotease group [10]. It demonstrates favorable clinical results following administration, with good or excellent rates of improvement in 70-90% of cases [4,5,11]. The safety profile is extensive, since certain disorders in the form of ecchymosis or even small skin openings at the injection site in the soft tissues of the hand have frequently been described. No cases of neurological or vascular involvement have been observed, since the predominant collagen in these structures is type IV. Serious complications, such as lysis of the tendon, have occasionally been described [12]. Since its approval for marketing in Europe in February 2011 [13], its use as an alternative for Dupuytren’s contracture (DC) has demonstrated the advantages of this non-invasive treatment (rapid recovery, low rate of complications and minimal skin alteration) and surgical treatments (eradication of the disease and a lower rate of recurrence). Its use has quickly spread throughout hand surgery units. The creation of infiltration and manipulation protocols in minor surgery operating rooms is allowing CCH infiltration to be gradually introduced as an alternative to fasciectomy, thus allowing for the optimization of both clinical and economic results for the center.

## Objectives

To analyze and compare the use of direct healthcare resources and the costs generated in the treatment of DC using two different techniques: subtotal fasciectomy and infiltration with Collagenase Clostridium Histolyticum (Xiapex®) in regular clinical practice in the Orthopedic and Traumatology Surgery (OTS) Department at the Hospital de Denia (Spain).

## Methods

### Study design

This is an observational, retrospective study using data from computerized clinical histories of two groups of patients followed-up in regular clinical practice starting at the time of the official opening of the center in February, 2009 and continuing to May, 2012: A) patients surgically treated using the subtotal fasciectomy technique on one or two digits; B) patients subjected to Collagenase Clostridium Histolyticum infiltration from the time of its commercialization in Spain in September, 2011 at the Orthopedic and Traumatology Surgery (OTS) Department of the Hospital de Denia (Spain) to May, 2012. For the inclusion of CCH patients in the surgery waiting list, two assumptions were drawn in terms of registry and billing according to the difference between a simple infiltration of soft joint tissue in the hand (coded in the ICD-9 as 82.96 other injection of locally-acting therapeutic substance into soft tissue of hand) with subsequent manipulation (ICD-9 93.26 manual rupture of joint adhesions) and a fasciectomy or fasciotomy (both coded in the ICD-9 with codes 82.12 and 82.35 respectively). Enzymatic Aponeurotomy (EA) is the name adopted for collagenase infiltration and the rupture of the cord is being referred to as Enzymatic Aponeurotomy Manipulation (EAM).

The authors declare that we have taken into account the ethical responsibilities included in these standards and that the procedures followed in the investigation are in accordance with the ethical standards of the committee on human experimentation or animal responsible (Ethics Committee of the research center) and in accordance with the World Medical Association and the Declaration of Helsinki.

### Inclusion criteria

All the patients included in this study were adults, diagnosed with DC and treated by means of one of two techniques: subtotal fasciectomy of one or two digits or CCH infiltration. The hospital’s pharmacy committee approved a protocol for treatment using Xiapex® with a maximum involvement of two digits per hand, usage according to the drug's technical sheet, treatment in the outpatient minor surgery unit and the construction of EA and EAM indications for invoicing; accordingly, patients with more than two digits affected in a single hand could not be treated with CCH and would be included in the FSC group. These patients were excluded from the study. Two patients had been previously treated (but not included in the study) with the aim of assessing the effectiveness of the July, 2011 treatment under the specific requirements of compassionate treatment before the official date of the drug's approval in Spain. The results obtained encouraged us to design the protocol and establish a generalized use for the drug. In order to establish CCH as the treatment of choice for DC in our center, we decided to compare our results with those previously obtained with surgery. Written informed consent was obtained from the patient for the publication of this report and any accompanying images.

For the review, all patients diagnosed with DC were selected by performing a computerized search of the hospital’s database. In the group with fasciectomy, all patients operated on for one or more digits from February, 2009 to May, 2012 were included. Likewise, the CCH group comprised patients treated from September, 2011 to May, 2012. Selection of treatment type was determined by the introduction of Xiapex® to the marketplace, the preferences and experiences of the surgeon, whether the surgeon had completed a training course for CCH infiltrations, the number of digits affected, and a clinical interview with the patient.

### Study variables

Variables studied in both groups were age, sex, number of digits affected and which ones, as well as degree of contracture according to affectation as classified by the British Society for Surgery of the Hand [14] (<30° light, 30-60° moderate and >60° severe) for the initial contracture and the results (0-5° excellent, 5-20° good and >20° unsatisfactory) with a residual flexion of 0-5° being considered to be excellent, as specified by Gilpin [15], 0-20° considered as good (the point used to define successful treatment) and 20° as unsatisfactory. Tubiana´s classification [16] of the patients is specified in Table 1. The healthcare resources included were rehabilitation sessions and OTS visits, as well as pre-operative preparation for the group of patients who were surgically treated.

**Table 1:** Demographic and clinical variables according to the type of treatment

**Variable**	**FSC (N = 48)**	**CCH (N = 43)**
Age (mean + SD)	65.9 (9.2)	65.1 (9.7)
Sex (male%)	83.3	88.4
Number of affected digits	64	50
Affected digits (average per patient)	1.33 (0.48)	1.16 (0.4)
Affectation	48 (100%)	43 (100%)
MCP	5 (10.4%)	7 (16.3%)
PIP	14 (29.2%)	12 (27.9%)
MCP + PIP	29 (60.4%)	24 (55.8%)
Contracture		
MCP	34 (70,8%)	31 (72,1%)
20-30	6 (17.6%)	8 (25.8%)
30-60	13 (38.2%)	10 (41.9%)
>60	15 (44.1%)	13 (41.9%)
PIP	43 (89,6%)	36 (83,7%)
20-30	9 (20.9%)	6 (16.6%)
30-60	20 (46.5%)	16 (44.4%)
>60	14 (32.5%)	14 (38.8%)
Tubiana’s classification [16] of treated radius	1 = 3	1 = 0
	2 = 28	2 = 20
	3 = 16	3 = 23
	4 = 1	4 = 0

No statistically significant differences between groups (p > 0.05).

### Costs

The approach selected was that of the National Health Service of Spain. Therefore, only the direct costs of healthcare were included, and indirect costs or expenditures associated with medical leave due to DC treatment were excluded. Table 2 shows all the different aspects of the study and their economic determination. The determination of costs in the FSC group was obtained using average surgical data measured by the center’s financial department, updated in June, 2012 for both groups. The valuation was conducted with respect to each of the processes in consideration of potential billing, since ours is a public hospital that is privately managed. Base parameters for billing take into account the average duration of surgical interventions, diagnosis according to the ICD-9 code and the basic costs applied to the use of the operating room with respect to an admission scheduled as major ambulatory surgery (surgery without admission in the major surgery operating room) or surgery with admission (surgery with an average stay of one night at the center) and others factors. The second group (CCH group) included the price of a CCH (Xiapex®) vial, as well as all costs derived from its use in a protocoled process of admission to the minor surgery unit (surgery without admission in the mini-operating room with local anesthesia). Both groups included pre- and post-interventions, pre-operative visits when required, hospital stays under the corresponding plans (surgery with admission, major ambulatory surgery or minor surgery), clinical and non-staff personnel involved in the process and other associated costs (Table 3).

**Table 2:** Unit costs (Euros) according to treatment type

**Variable**	**CCH**	**FSC**
Pre-operative		66.02
Anesthesia consultation		59.02
First OTS visit	56.03	56.03
Costs associated with surgery/treatment	748.51	904.05
2 Surgeons		229.34
1 Anesthetist		134.15
Non-staff personnel, field and circulating		180.62
Support personnel (assistants)		8.38
Specific surgical materials		327.47
Pharmacy		24,09
Mini-operating room (surgeon + nurse)*	22.21	
Injection and dressing materials	1.30	
Collagenase clostridium histolyticum (Xiapex®)	725	
Hospitalization		267.48
Major ambulatory surgery (MAS)		121.32
Minor ambulatory surgery	77.28	
Subsequent OTS visits (average, mean cost)	1.23; 51.68	1.98; 83.20
Hospital care (average, mean cost)	0.74; 19.02	2.00; 51.42
Rehabilitation (frequency, number of sessions, mean cost)		29%; 18.4; 473.83

OTS: Orthopedics and Traumatology Surgery, MAS: Major Ambulatory Surgery.

*Subjective cost of one working day of 6 hours for two consecutive days by one surgeon, resulting in €16.21 per patient. The same scale is applied to a single nurse present in the mini-operating room with a cost of €6.00 per patient without any assistants.

**Table 3:** Costs of different items (euros) according to the type of treatment

	**FSC**	**Total FSC**	**CCH**	**Total CCH**
Costs prior to surgery	181.07		56.03	
Operating room costs	904.05	1085.12	748.51	804.54
Post-surgical costs (care + visits)	134.62	1219.74	70.70	875.24
MAS without RHB	121.32	1341.06		
Admission without RHB	267.48	1487.22		
RHB	473.83			
MAS with RHB		1814.89		
Admission with RHB		1961.05		
Minor ambulatory surgery			77.28	952.52

FSC: Fasciectomy; CCH: Collagenase Clostridium Histolyticum; MAS: Major Ambulatory Surgery; RHB: Rehabilitation. Minor ambulatory surgery requires no admission and is performed in a mini-operating room.

This cost was expressed in terms of average cost per patient (€, June, 2012) for the main variables, in total, for each study group, as well as a comparison (differences) among groups. Discount rates for annual upgrade costs were not applicable.

### Data confidentiality

Prior to analysis, steps were taken to assure data confidentiality. The study was classified by the Spanish Drug and Health Products Agency (*Agencia Española de Medicamentos y Productos Sanitarios, Non-EPA*) and subsequently approved by the center’s Ethics Committee for Clinical Research.

## Results

A total of 91 DC patients were included, 48 (52.75%) in the FSC group and 43 (47.25%) in the group treated using CCH.

The group subject to subtotal fasciectomy surgery included a total of 48 patients with a mean age of 65.9 (9.2) years (50–80 range) and a mean of 1.33 (0.48) digits affected. The CCH group comprised 43 patients aged 65.1 (9.7) years (48–87 range) and a mean of 1.16 (0.4) digits affected. There were no statistically significant differences between the baseline characteristics of the treatment groups.

All patients made a preliminary visit to the OTS department, where their conditions were assessed and a basic clinical history was created: clinical history and data of interest were collected and patients were informed of the procedure to be undertaken.

All patients treated with FSC were requested to complete a pre-operative test (blood test with biochemistry, hemogram and coagulation, electrocardiogram, and chest radiography if over the age of 60). Next, they were scheduled for a pre-anesthesia visit for surgical evaluation. The CCH group had no pre-operative tests.

In the FSC group, a total of 48 patients were treated, 5 (10%) with isolated MCP involvement, 14 (29%) with single alteration of the PIP and 29 (60%) with combined affectation. Patients treated with CCH showed a single involvement in the MCP in 7 (16%) cases and in the PIP in 12 cases (28%). Combined cases added up to 24 (56%). Fingers affected were: 5^th^ finger in 20 cases for the FSC group and 25 for the CCH group, 4^th^ finger in 21 and 17 respectively, 3^rd^ finger in 4 and 1, and in the FSC group, one 2^nd^ finger and two 1^st^ fingers were also treated. A total of 29 infiltrations with collagenase were performed at the MCP joint (14 in 5^th^ finger, 14 in 4^th^ finger, and 1 in 3^rd^ finger) and 14 at the PIP joint (11 in the 5^th^ finger and 3 in the 4^th^). Table 1 presents the data corresponding to initial involvement.

In the FSC group, 39 (81.3%) of the patients were treated under an outpatient hospitalization plan (major ambulatory surgery) while 9 (18.7%) required hospital admission for a day due to medical criteria (patients with multiple pathologies, those who take oral anti-coagulants and those with long-lasting brachial plexus anesthesia or total anesthesia in surgeries performed in the afternoon). In the CCH group, all patients were treated under an outpatient plan (minor surgery).

In the FSC group, the operating room had two surgeons, one anesthetist, non-staff healthcare personnel (field nurses and circulating nurses), support staff (nurse’s aides), materials for both general surgical procedures (gauzes, pads…) and those specific to that surgery (hand surgery box), anesthesia and pharmacy-derived costs. Operation room structural expenses, such as power and water, were not included, since this room was considered functional for this activity and for any other for both groups. Taking this data into account, the costs of the surgical intervention come to €904, with the admission plan (hospitalization or outpatient) to be added.

For the CCH infiltration, all patients went to the center under an outpatient plan for minor surgery. In order to optimize resources, patients were gathered into groups (in three surgical sessions of 14, 16 and 13 subjects; average of 14.3). Two consecutive days are required for this pharmacological treatment: the first for the infiltration and the second for manipulation of the cord. This allowed a record of the patient’s drugs to be drawn up by the pharmacy department. Since the procedure was standardized, it allowed for simultaneous invoicing of the product, while maintaining the cold chain supply from the pharmacy. For the patient, it also had the advantage of his/her condition being monitored in the non-admissions surgical unit (NASU) with the assistance of non-staff members and nurses in case complications should arise. Both the infiltrations and the manipulations took place in a mini-operating room instead of at outpatient services. Expenses arising from the CCH treatment stemmed from the cost of the vial of product, non-staff personnel (only a trauma doctor and a nurse were present, with no anesthetist or other support staff needed) and materials for dressings and injection.

After the surgery, patients were examined and their wounds dressed in the hospital consultation office. The date for the first follow-up visit for patients in the FSC group was 4–5 days after the intervention, coinciding with the first day of visits by the authors for the assessment of the surgical wounds. In the CCH group with skin opening, phlyctena with hemorrhagic content or any type of complication, the first visit took place after those same 4–5 days. If there were no problems, patients were asked to return after 1, 3 and 6 months in order to assess the functional results of the treatment. The average number of medical examinations was 2 and the number of hospital dressings was also 2 in the FSC group. A total of 14 patients required visits to the rehabilitation department, with an average of 18.43 physiotherapy sessions (range 10–45) involving an average cost of €473.

In the CCH group, the evolution of the CORD I and CORD II [15,17] study protocols were followed, with subsequent control visits at 3, 6, and 12 months (mean of 1.23 visits). The infiltration of a different digit or hand was considered a new starting point, rather than a subsequent visit. When the study was performed, only the check-ups for months 3 and 6 had been completed for certain patients. The rate of hospital care in terms of skin openings or any other complication was 0.74.

The average correction of the contracture with CCH infiltration at the MCP joint in an isolated manner was 88% of the initial contracture with an average correction of 42°. 4 cases showed an excellent result (0-5° contracture), 2 a good result (5-20°) and 1 unsatisfactory. Regarding PIP, the average correction was 32° (52%) with 4 cases considered to have an excellent result, 1 good and 7 unsatisfactory. In those patients in which infiltration was performed at the MCP or PIP with involvement of another joint in the same digit (infiltration was made in the most affected), the average correction was 40° (87%) in the MCP joint (17 excellent results, 5 good and 2 unsatisfactory) and 29° (67%) for the PIP (9 excellent, 8 good and 7 unsatisfactory results). Table 4 shows comparative data of the results among groups.

**Table 4:** Results of the treatment in both groups

		**CCH**						**FSC**		
**Total**		**Def Ext med**	**N° patients**	**Mean (%)**	**Range**	**Average correction°**	**Range**	**Def ext med**	**N° patients**	**Mean (%)**
MCF		12	31	77	0-100	36	0-94	10	34	91,4
	0-5	2,3	21	99		43		4,1	19	97,4
	5-20	12	6	77		44		7	11	92,3
	>20	57	4	25		19		26	4	85,6
IFP		21	31	57	0-100	27	0-80	17	43	64,4
	0-5	1,3	9	98		41		4,6	17	93,1
	5-20	13	10	63		30		14	19	70,1
	>20	49	12	26		16		30	7	48,7

The CCH group had a greater complication rate (2%) due to a patient with intense hyperesthesia in the puncture site. This was intense and continuous and did not ease with any medical or physiotherapeutic treatment type, and a dermofasciectomy was performed, which cured the condition [15]. A total of 3 early recurrences (7%) were observed and could be due to defects in the drug's administration technique, since they took place in the first batch of infiltrations, and their results, although unsatisfactory, were included in the result statistics. In one case, the presence of air in the barrel of the syringe caused the administration of a lower medication dose to the patient, which was probably the cause of treatment failure. The other two cases were unsatisfactory infiltrations at the ulnar flanges of the 5^th^ finger. In both cases, surgery was performed by means of subtotal fasciectomy; in one case the patient did not accept further treatment. All problems with CCH were detected at the time of the first follow-up visit one month after intervention, and reoccurrences saw the patients’ conditions brought back to the same degree as before the infiltration. Thus, we conclude that it was a probably technical problem which affected only the first cases treated. In the FSC group, greater complications were found in 9 (20%) patients: 1 digital nerve lesion, 3 cases of infection, 2 cases of stiffness solved with intensive physiotherapy, 2 cases of CRPS (complex regional pain syndrome) and 1 amputation.

**Figure 1:**
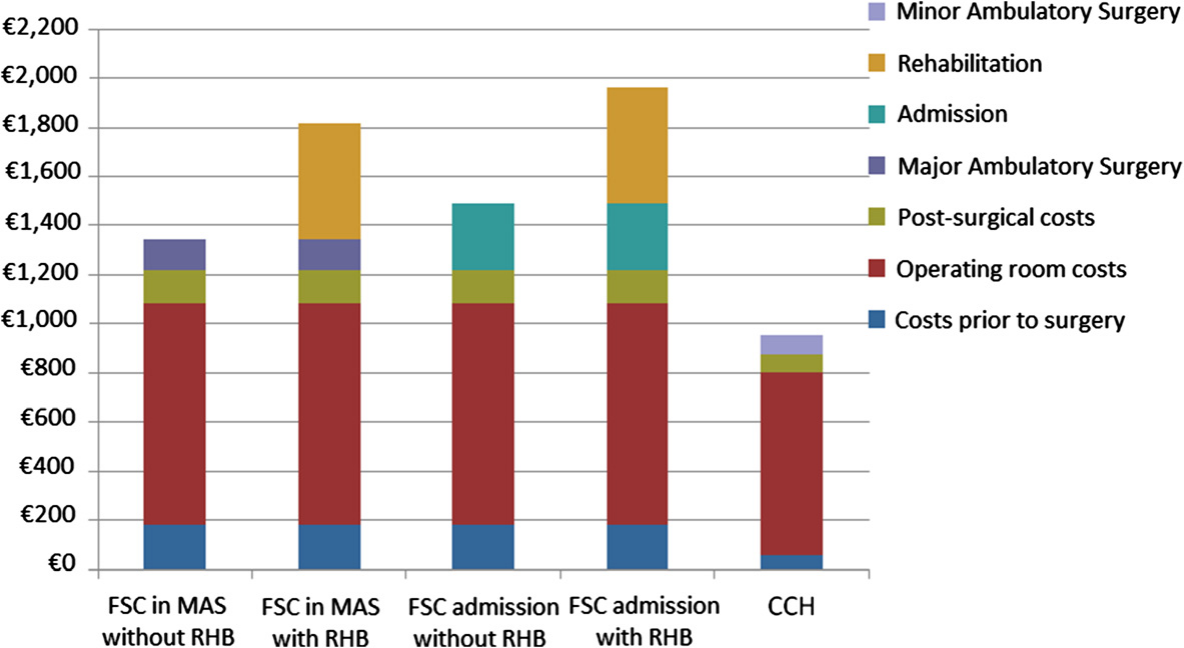
Cumulative costs of different items according to the type of treatment and admission.

Finally, comparing the full costs of both treatments, a total savings of €388 is estimated in favor of CCH treatment in the best case scenario for FSC (patient under MAS plan with no need for physiotherapy) and €1,008 in the worst case scenario for FSC (patient admitted to hospital needing subsequent physiotherapy), thus signifying a total savings of 29% and 51.5%, respectively (Figure 1).

## Discussion

There are no current studies assessing the costs associated with DC treatment in Spain that include CCH treatment. This is the first study comparing the use of resources and expenses related to surgical treatment with fasciectomy versus CCH infiltration under conditions of regular clinical practice. De Salas-Cansado [18] compared fasciectomy procedures at three primary hospitals in Spain. The average total cost per treatment was €2,250. Costs varied according to hospital and admission plan, but were quite similar to our conclusions: patients admitted had a cost of €2,467 compared to €1,961 in our study, and ambulatory patients had a cost of €1,703 compared to €1,815 in our study. The difference lay primarily in the cost of the hospital stay, since costs derived exclusively from the surgery itself were also quite similar (€1,074 compared to €904, respectively). An increase in the number of ambulatory patients, especially in the mild stages of DC and those with few co-morbidities, is proposed in this article. In our case, the use of local anesthesia and the presence of a recovery room adjacent to the mini-operating room where CCH treatments took place allowed us to include all patients in the minor surgery plan, as we were able to maintain adequate monitoring of patients after infiltration and manipulation, thus minimizing costs and resources.

Chen [19] established a cost-utility analysis comparing several techniques based on quality-adjusted life years (QALY) by creating several possible situations. The study concluded that CCH is a cost-effective treatment with a treatment cost of less than $945 (€730) (in Spain, the current price of Xiapex© is €725 per vial) with associated costs of $122 (€94.20) for the injection, and a resulting total of €824, which is quite similar to our estimate for the treatments in which only one injection is required and the cost of subsequent examinations are not included (we did include these). Therefore, our results are more economical.

The most extensive population study may be that presented by Gerber [20], which included more than 60,000 NHS cases. This study did not consider the use of CCH. Among the registered treatment options, fasciectomy was used in 91% of cases for the treatment of DC. The average established costs were £2,885 (€3,579) for ambulatory patients and £3,534 (€4,384) for admitted patients. There was also a difference with the price of digital palmar fasciectomy, which was more expensive. Although estimated costs varied according to the hospital and the condition of the patient, they are significantly higher than those represented in our geographical area. In France [11], the cost of fasciectomy has been established only for admitted patients over the age of 45. An extended mean stay [2.3 (1.9) days], differences in costs between public and private sectors, and changes in costs since the article’s date of publication (2005) nullify the validity of these findings with respect to the parameters we are now studying. In addition, this author overestimated costs, since ambulatory patients were not considered. The price of percutaneous fasciectomy was unusual, since it was priced at €37 for one hand and €65 for two hands.

Even though needle fasciectomy is by far the cheapest treatment, recurrence rates, varying after 5 years from 50-85% [21,22], make this treatment type unacceptable, since education and physiotherapy costs (frequently not offset due to the quick recurrence of the disease) have to be added to the advantages of a minimally aggressive procedure. In one analysis, Chen [19] conducted an additional review of the recurrence rate according to the technique used for the treatment of DC and concluded that needle fasciotomy had a recurrence rate of 60%, fasciectomies 30% and CCH infiltration only 15% for a complete treatment of 3 injections. A review of the literature provides recurrence rates between 15 and 46% for surgery, 0 to 75% for CCH, 12 to 65% for needle fasciotomy, 12% for dermofasciectomy, and 23% for skeletal traction, with great variability regarding the time of evolution and no consensus as to the definition of recurrence [6,14,15,21-27].

For our regional healthcare system, the government for the region of Valencia for the 2012 [28] fiscal year established prices for hospital processes according to DRG (diagnosis-related groups). These were used for correlating different types of patients treated in a hospital with the costs incurred for their care. There was no specific DRG for DC, although it was normally included in DRG228 (major thumb or joint procedures or other hand or wrist procedures) or DRG229 (hand or wrist procedures, except major joint procedures). The amounts allocated for both codes were €3,501.59 and €2,799.03, respectively. These were much higher than our findings and about 290-360% more expensive than infiltration with CCH at our center. Currently, Enzymatic Aponeurotomy is not included in the treatment with CCH, nor is CCH infiltration specified, although in the rehabilitation section, a cost of €677.96 is established for infiltration with botulinum toxin (PR1201), which would be the most approximate option. The price of the vial to be infiltrated and the subsequent manipulation, as concomitant therapy, should be added on.

For the treatment of DC, the use of CCH in surgery units without admission allows for a combination of treatment efficacy with minimal aggression and hospitalization. Results are good with AVS after infiltration at a one month clinical revision, as is patient satisfaction. In addition, CCH optimizes healthcare resources, at the present time a scarce commodity. Our study presents the costs in a detailed and exhaustive manner for both alternatives assessed, since aside from hospital expenses, it also includes all costs for follow-up visits and post-operative rehabilitation. However, several limitations must be noted. The first limitation is related to the progress of treatment with CCH, because even though 8-year progress studies [24] are available, the number of cases involved does not allow us to draw conclusions regarding the long-term progress of these patients after fasciectomy. Rates of early recurrence and other types of disorders (such as skin retractions) should also be assessed. These might show unsatisfactory results over the short term. Another limitation is related to costs, because the inflation rate in Spain has been 2.76% from June, 2012 to the present time, so that all costs of the process have increased to this extent. The inflation rate in the rest of the Euro area has been only 2.29% for the same period and this will affect values when comparing our data with those of other countries. The price per dose of Xiapex® has not changed.

With respect to our economic assessment, it is estimated per process, that is, per infiltration. Therefore, in patients for whom the treatment of a cord requires two or three infiltrations, the price is doubled and tripled, respectively, since this scenario is not considered a recurrence. Likewise, patients with two or even three digits affected that are resolved using a single infiltration should be considered differently, because the final cost of the treatment is then reduced when compared to one hand, since treatment with CCH is initially conceived to be used in only one joint per treatment. The results in our study indicate a rate of those affected of 1.16 for the CCH and 1.33 with surgery (p > 0.05). That entails a reduction, in many cases, of 2 or even 3 digits with a single Xiapex^©^ infiltration, especially in cords at the metacarpophalangeal (MCP) joint that are of natatory or Y types.

The results obtained with CCH are clinically similar to those of the FSC group. Fewer major complications have been observed in the CCH group at midterm, and infiltration with collagenase in one finger, on many occasions, treats two joints. The explanation for not meeting the criteria in the technical sheet with 3 vials is two-fold in our environment. First of all, it is related to patients; the infiltration is frequently painful and performed with no anesthesia. When patients experience a significant improvement of the contracture, sufficient for performing both work and daily activities, they sometimes reject further treatment, and do not obtain the good or excellent correction that might have been achieved. This is the case with those patients suffering severe MCP and PIP involvement, in which MCP is completely corrected and 30° remain, for example, of PIP flexion. The second reason is related to infrastructure; it is very difficult to schedule and carry out a second infiltration just one month after the first one for those patients who did not achieve a satisfactory outcome, and practically impossible to schedule a third one.

Our results with the use of CCH are similar to those of Hurst [17]. Clinical improvement in the second end point is somewhat lower in our results (74% in our study as opposed to 84.7% in the Hurst study). On the other hand, ROM improvement is similar (an average of 36° in our study compared to 41° in the Hurst study for the MCP and an average of 27° versus 29° respectively in the PIP joint). The minor correction of 5 degrees for both the MCF and the IFP is lower in our study, which may be due to several factors: the use of only one infiltration, the initial learning curve, or the presence of very marked contractures.

Regarding the recurrence rate, it is too soon to determine the problems that may appear in the long term for those patients treated in the CCH group. The average follow up time in the study does not allow us to make valid predictions as to the recurrence rate for these patients, although the results seem promising. Study data are upfront costs in CD. Long term evolution of CCH treatment is uncertain and the recurrence rate unknown, *although* the systematic review conducted by Chen [29] seems to indicate that the use of CCH has a lower rate of recurrence than does FSC. At the present time, we consider CCH as a definitive treatment or as a treatment that may reduce the number of patients needing surgery over time [30], especially for those with lower degrees of contracture and isolated involvement of the MCP joint.

## Conclusions

In the short term, treatment with CCH infiltration reflects a 29 to 51.5% decrease in healthcare costs (€388 - €1,008) and in use of resources in comparison with the treatment of choice for DC: fasciectomy. Assigning the codes Enzymatic Aponeurotomy (EA) and Enzymatic Aponeurotomy Manipulation (EAM) to the process allows for registering and monitoring both processes and patients for adequate treatment and future procedures.

## Competing interests

The authors declare that they have no competing interests.

## Authors’ contributions

RSC has made substantial contributions to conception, design, data collection, data analysis and interpretation of and writing of the manuscript; NFF to conception, design, data collection, data analysis and interpretation and providing a critical review of the manuscript. All authors read and approved the final manuscript.

## Pre-publication history

The pre-publication history for this paper can be accessed here:

http://www.biomedcentral.com/1471-2474/14/293/prepub
